# The Future of Biobanking: What Is Next?

**DOI:** 10.3390/biotech9040023

**Published:** 2020-11-23

**Authors:** Luciana Caenazzo, Pamela Tozzo

**Affiliations:** Laboratory of Forensic Genetics, Department of Molecular Medicine, University of Padova, 35121 Padova, Italy; pamela.tozzo@unipd.it

**Keywords:** biobank, biospecimen, harmonization, regulation

## Abstract

Biobanks are an extraordinary tool for research and scientific progress. Since their origin, the debate on the main technical, regulatory and ethical aspects has not stopped. The future of biobanks should take into account many factors: the need to improve the technical standards of collection, conservation and use of the sample, the usefulness of achieving forms of harmonization and common governance, the improvement of biobank networks, including through public–private partnerships and improving the sustainability of these infrastructures.

## 1. Introduction

Biobanks are an extraordinary tool, which for decades have been progressively creating new research platforms and new possibilities to learn more about the function of living systems in physiological and pathological conditions both acute and chronic [1]. The future of human biobanks in the era of “new biology” can lead to encouraging scenarios for understanding the more complex mechanisms of biodiversity and the physiological and pathological mechanisms that underlie the state of health of human beings [2]. New research frontiers concern studies on longevity, on predictors of human behavior, on the refinement of -omics techniques [3]. An endless amount of biological samples and associated data are kept and will be stored in the future in various types of biobanks around the world, and this potential should be exploited, ensuring the satisfaction of new infrastructural needs and the application of new and more effective technologies for research [4]. Biobanks were initially developed in relation to a specific research question and designed for that specific aim. Even considering only biobanks of human specimens, there are several types of biobanks. The two major subtypes are: population-based biobanks and disease-oriented biobanks. The conditions for the establishment, procurement and use of samples in these two different types of biobanks are very different, given that in disease-oriented biobanks samples are collected mainly from residual materials taken from patients for diagnosis or treatment; in the other case, instead, samples are collected from healthy participants. It is clear that the new challenges posed by the future developments of biobanks—as outlined in this commentary—can be declined and, probably even resolved, in different ways, depending on whether one or the other type of sample collection is referred to.

The use of large quantities of human biospecimens (blood, saliva, biological tissues and fluids and nucleic acids) and data associated with them through the application of new technologies cannot ignore the integration of many different skills: biological, medical, biochemical, biotechnological, bioinformatics, epidemiological, engineering and, last but not least, economic skills, in order to ensure the highest productivity and profitability of these large infrastructures.

Biobanks involve the collection, storage, use and distribution of biological samples for research purposes. This is an activity that currently involves the vast majority of hospitals and biomedical research structures and constitutes the pillar on which to build an effective, efficient and modern research system. Originally, the collection of biological samples for research stemmed from the conservation of waste/residual material from biological samples taken for diagnostic and/or therapeutic reasons [5]. Over time, these initially sporadic and wholly improvised collections have begun to become increasingly popular, prompting scientists to recognize the need for more uniform, more coordinated and more organized collection-and-use systems in order to guarantee excellent research in the genomic and post-genomic era [6]. This has pushed biomedical research and assistance structures to integrate with private structures, starting a path of harmonization and common governance of biobanks [7].

The progress of available technologies and the need to have research results that are increasingly shared and comparable has made it mandatory to start a phase of implementation of biobanks, in terms of samples and data, projected towards the future, which takes into account the new requirements of standardization, governance, of public–private partnerships, guaranteeing high quality standards and corporate organization of the same.

The future of biobanks is already present, and the areas of development in the world of biobanks are many.

## 2. Advances in Technical Storage

The new advances in the field of biobanks strictly depend on the improvement and implementation of increasingly sophisticated, targeted and standardized techniques for the collection and storage of samples. The quality of the samples, the quantity of available material, the possibility of identifying new sources of collection and the efforts to harmonize the technical aspects of the collection are crucial elements for creating useful and productive biobanks [8]. Traditionally, blood, urine and (frozen) tissues have been the primary sources of implementation for biobanks; more recently, the habit of collecting new samples with high information potential has spread, such as circulating tumor cells, liquid biopsies and free-circulating DNA. It is evident that these new types of material require high standards of both sampling and storage, as well as shared protocols to ensure the maintenance of their quality and therefore the sustainability of biobanks that feed on these samples [9].

It is thus necessary to integrate the more traditional technical principles with which biobanks were created and developed, with different scientific foundations concerning the fields of cryobiology, biomedical engineering and biotechnologies since the main objective of these new biobanks is to maintain the quality of samples by improving the sustainability of the entire infrastructure [10].

Tissues and samples used in biobanks should be able to maintain their morphological and functional characteristics, even after the application of conservation methods, in order to make the results obtained reproducible and comparable but also to be able to apply translational research more effectively in many areas, especially in oncology, gene therapy and personalized medicine. However, there remain problems related to the conservation of the samples, such as the loss of some cellular functions under certain storage conditions and the need to use some animal components for preservation that could interfere with human cellular functions, activation or inactivation, in certain conditions, of some cellular mechanisms (signal transduction, gene modulation and protein expression) that would render the samples unusable [11].

Today, it is therefore necessary to have readily available samples of different types in adequate quantities, which do not lose their morpho-functional characteristics after storage. At the same time, it is useful to ensure preservation mechanisms with rational costs and with sustainable technologies, which do not excessively affect the costs and management of the infrastructure, through a capillary collection system alongside centralized storage and conservation structures. This could make it possible to guarantee large numbers of samples and their variability, also ensuring high quality standards and, possibly, cost containment.

## 3. Harmonization and Standardization

Harmonization and standardization are crucial elements in the development and future sustainability of biobanks [12].

Many efforts have been put in place in both the USA and in Europe to find collaborative dialogue platforms aimed at the drafting and application of shared protocols and common rules that mainly concern the procurement of samples, their collection and storage, treatment data related to the samples and the exchange of material and information, as well as the administrative and financial management of the infrastructures connected to biobanks [13]. Equally important, alongside the technical harmonization of biobanks, must be the harmonization of regulatory and, above all, ethical aspects related to biobanks: consent of the participants, confidentiality of data and anonymization of samples and return of results.

Better harmonization can lead to important results, such as obtaining an orderly and multicenter collection of data and samples, creating homogeneous groups of patients with very large and well-described case histories and the possibility of creating studies on large cohorts and sub-cohorts of patients. The application of uniform standards could also ensure greater long-term safety in the management, storage and distribution of samples, through the use of innovative and common systems of logistics management and traceability of samples.

A fragmentation of protocols and guidelines is certainly not functional to the new challenges posed by biomedical research: we need comparable research platforms, which produce reproducible results and, above all, a common effort should be put in place, in order to analyze many samples in different infrastructures, making research results more usable and more effective in daily clinical practice.

## 4. Biobanks Networking

Currently, basic and translational research cannot do without the use of very advanced molecular biology techniques for which it is necessary to have suitable samples in large quantities. It is evident that the availability of many samples that comply with adequacy standards may not be guaranteed by a single biobank, so it is necessary that several infrastructures coordinate with each other in order to exchange samples and data, obviously favoring collaboration both at a national and international level. The networking of biobanks allows for multiple collection and, sometimes, storage sites, which converge into a single platform for sharing and exchanging resources [14].

Many biobanks participate in activities that have been organized as partnerships that have given rise to national and international networks focused on specific purposes [15].

The research developed thanks to the biobank networks will benefit from the advantages that derive from the sharing and exchange of samples, while the researchers belonging to structures that are outside these networks may not have the same benefits. Networking of biobanks within academic facilities could push non-academic researchers, private research facilities and pharmaceutical and biotechnology industries to find samples from other sources, such as commercial biobanks. It may be necessary to improve the comparison and collaboration between public and private structures, with the sole aim of seeing improved research quality standards [16].

## 5. Biobanks’ Sustainability

The economic sustainability of biobanks has become, especially recently, a central theme [17,18]. As the global economic situation has deteriorated, funding for most public biobanks has shrunk, prompting biobank organizations to seek to implement strategies to make these infrastructures more sustainable. Various strategies have been proposed to try to contain costs (adoption of automation, avoiding the underutilization of bio-specimens, increasing business administration studies in the biobanks, etc.), while improving the development of partnerships with pharmaceutical companies and biotechnological industries or promoting the provision of services for a fee [10,19,20].

These strategies have been only partially effective since cultural and regulatory issues often prevent biobanks from being self-sustaining. Biobanks are infrastructures that, in recent years, have been involved in transformative processes aimed at the construction of dedicated business plans and organization from a business and commercial point of view. The aim should be to make these infrastructures more efficient and more sustainable. The organizations that support the establishment of biobanks have begun to recognize the need to improve the level of professionalism in management, in the management and training of personnel, in the procurement and sustainability of structures, in a more corporate and industrial perspective on biobanks. Indeed, from a cultural point of view, it is often difficult to make even the management of the biobank understand how high the costs of collecting, storing and maintaining samples are. On the other hand, even at the level of local and national regulations, there is often a ban on profiting from the use of biological samples. Despite these obstacles, there is the possibility of establishing a precise plan for both structural and financial reorganization, which will make it possible to make a biobank more sustainable. The basic steps are: to hire expert consultants in finance and business organization and educate them on the peculiarities of a biobank; analyze costs and improve the relationship between cost and effectiveness; maximize the availability and usability of samples limiting the sample waste; develop a business model that identifies potential users by aligning the supply of samples to the needs of stakeholders to make a biobank more sustainable in the long term [21].

## 6. The Future of Data Protection

In the future of biobanks, there is also, undoubtedly, the need for a homogenous and shared management of the return of results and incidental findings.

In the context of biobanks, informed consent to the collection and use of samples must ensure that patients and donors are fully informed on the methods of collecting, storing and using the samples, on issues related to confidentiality, in particular the risk of disclosure of the donor’s identity, and the potential for commercial use of the samples [22,23].

There are four measures that have been considered crucial by the IBC to protect the rights of individuals, having acknowledged that future research on human health cannot ignore the use and management of big data: governance, education, capacity building and benefit sharing [24]. These aspects, also in relation to data protection, cannot be forgotten in the management of biobanks. The objective of the IBC is to stimulate debate and discussion on an international scale between various stakeholders (patients, participants and citizens in general) and also in the context of institutional and political governance and research centers, both in government and international agencies (WHO, UNESCO, UN).

In particular, the application of shared ethical and regulatory standards, both nationally and internationally, is still far from being achieved, and regulatory and ethical harmonization is even more distant than organizational and structural harmonization. There are numerous issues, some more traditionally linked to biobanks, others newer, that must also be addressed in the future of biobanks: material transfer agreements, intellectual property, access to samples and data, ownership and custody of data and samples, return of results and incidental findings.

## 7. Conclusions

The future of biobanks is already here. The need for technological advancement can aid in the development of research. Nevertheless, it is necessary that the management of biobanks be up to date from both a financial level and, above all, from an ethical and regulatory point of view. This discussion on biobanks is not outdated: the strength of biobanks lies precisely in being able to always stoke new and exciting debates despite the inexorable passage of time.
